# Optimising Educational Practices in Simulation‐Based Nursing Education: Results of A National Survey

**DOI:** 10.1002/nop2.70172

**Published:** 2025-04-23

**Authors:** Mohammad Alsadi, Mohammad R. Alosta, Elham H. Othman, Hayyaf Obiedallah, Abdulqadir J. Nashwan

**Affiliations:** ^1^ School of Nursing (SN) German Jordanian University (GJU) Amman Jordan; ^2^ Faculty of Nursing Zarqa University Zarqa Jordan; ^3^ Faculty of Nursing Applied Science Private University Amman Jordan; ^4^ Nursing Department Hamad Medical Corporation Doha Qatar

**Keywords:** educational practices, Jordan, national survey, nursing students, simulation design

## Abstract

**Introduction:**

Simulation‐based education is recommended in nursing education. In the same stream, different educational practices are utilised using different simulation designs to optimise the learning outcomes. The current study aims to evaluate the better simulation designs in undergraduate nursing education and the associated “better fit” educational practices.

**Methods:**

Descriptive, correlational design using a self‐reported online questionnaire.

**Results:**

A total of 966 undergraduate nursing students at 16 universities in Jordan participated in the survey. Nursing students perceive “support” during the simulation‐based educational activity, clear “objective and information,” and high “fidelity” as the important dimensions of simulation designs. An “active learning” experience was considered the most important educational practice.

**Conclusions:**

The simulation laboratories need to support students, clarify the objectives, and provide needed information. High levels of fidelity are also needed, coupled with interventions to support students' active learning experiences.

**Patient or Public Contribution:**

No Patient or Public Contribution.

## 1 Introduction

Simulation‐based education is highly recommended for healthcare professionals' education and is considered essential in preparing nursing students for clinical practice (Cant and Cooper 2017; Tawalbeh 2020). Aggravated by the spread of COVID‐19, more than 300 million students worldwide had their education interrupted, including undergraduate students with different specialties and learning requirements (Unicef 2021). Nursing and medical students are more affected by the pandemics due to the enforced access restrictions to clinical training settings; providing limited clinical experience can impart technical knowledge that is inadequate to prepare nurses for the complexities of the workplace (Nabolsi et al. 2021; Tawalbeh 2020). Therefore, nursing schools were required to adapt and change the educational techniques to continue preparing students for the real world of healthcare and facilitate their transition into the role of independent caregivers (Escudero et al. 2019; Masuda et al. 2022).

To act proactively in finding alternative options to back up the routine educational approaches, reliance on technology (i.e., simulation‐based education) was adopted among the main alternatives (Masuda et al. 2022). Simulation‐based education provides realistic clinical situations in a safe environment for learners, enhances and improves theoretical and clinical learning skills, and controls the tasks provided to students. It can be started with easier tasks and then proceed in difficulty, maintaining the excellence of students' knowledge, skills, and clinical experience (Cant and Cooper 2017; So et al. 2019).

Simulation‐based education has been a pre‐qualifying educational method in undergraduate nursing education for over a century (Aebersold 2018). It is defined as “a technique that creates a situation or environment to allow persons to experience a representation of a real event for practice, learning, evaluation, testing, or to gain understanding of systems or human actions” (Society for Simulation in Healthcare 2022, 14). Not merely handling mannequins, simulation‐based education in nursing encompasses a wide range of simulation designs ranging from Low‐Fidelity Simulation (LFS), which can be described as having a lower ability to mimic real‐life situations, then to High‐Fidelity Simulation (HFS), which has advanced technology and tools to mimic real‐life scenarios (Hanshaw and Dickerson 2020). Simulation‐based education aims to bridge the gap between theory and practice through the involved interactive educational practices (Chang et al. 2021). It is considered a useful, beneficial, and effective educational approach that provides the appropriate setting for nursing clinical training. Students are immersed in ‘real‐life’ patient scenarios, which test their nursing skills while engaging their emotions, which is more associated with high‐fidelity designs (Shorey and Ng 2021).

Although simulation‐based education was adopted at many nursing education schools worldwide and is considered a powerful teaching strategy, questions continue to arise about the suitability of simulation experiences to educate nursing students. Simulation implementation as a teaching strategy must be based on pedagogical principles and well‐defined educational practices (Jeffries 2005). For instance, educators need to know more about the best teaching and learning practices with the simulation that will contribute to the best positive educational outcomes (Masuda et al. 2022; Tawalbeh 2020). In the literature, studies that examine nursing students' best educational practices using different simulation designs are yet to be conclusive in addition to being highly based on situational factors (e.g., availability of advanced technology). Moreover, limited studies guide designing, implementing, and evaluating simulations in nursing (Ross and Atkinson 2020; Tawalbeh 2020).

The current study evaluated the simulation designs utilised and the associated educational practices implemented in nursing education in Jordan. It aims to identify the preferred simulation design and educational practices as perceived by nursing students.

## 2 Methods

### 2.1 Design

This survey was conducted nationally in Jordan using a descriptive, correlational design. A self‐report questionnaire assessed undergraduate nursing students' perceptions of the simulation designs and the associated educational practices. The questionnaire was built online using Google Forms.

### 2.2 Setting and Sampling

The target population was all undergraduate nursing students in Jordan. At the time of the study in 2022, 16 public and eight private universities offered undergraduate bachelor's degrees in nursing in Jordan. All 16 universities were approached and agreed to participate in the study as they all contain simulation‐based education in their curricula. The participating schools of nursing have a total of approximately 7000 undergraduate nursing students. All undergraduate nursing students who received at least one simulation educational course were invited to participate in the study. A convenient sampling design was used as the announcement to participate in the study was posted on the announcement boards at nursing schools and on the online announcement platforms for each school. The nursing deans and heads of academic nursing departments shared the link to the online questionnaire. The data collection took place between October and December 2022. To calculate the minimum needed sample size, Slovins' formula was used, which is based on the population size, level of Confidence Interval (CI), and margin of error (*α*) (Bowers 2019). As the population was estimated to be 7000 nursing students, and the cI was set at 95% with the margin of error to be *α* = 0.05, the minimum needed sample was 365 students.

### 2.3 Instruments

The online data collection consisted of three sections: demographics, the Simulation Design Scale (SDS), and the educational practices questionnaire (EPQ).

The demographics sections asked the students to report their age (in years), gender, academic year (first, second, third, and fourth year), and enrolment type (new admission from secondary school or LPN to RN program). Moreover, this section asked the students to rate their computer skills using a single‐item scale ranging from “weak” computer skills, going through “average” skills, and “excellent” computer skills, which was developed by Staggers (1994). Then, students were asked if they thought their computer skills affected their experience in simulation‐based education (Yes, No).

The Educational Practices Questionnaire (EPQ) developed by the National League for Nursing (NLN) consists of 16 items assessing the participants' views of best educational practices in simulation and their importance. Ten items examine learners' attitudes and opportunities toward “active learning,” and six items examine students' “collaboration,” “diverse ways of learning,” and “expectations” presented during simulation. Response options for each item using a Likert‐style scale are similar to those in SDS (Jeffries and Rizzolo 2006). Cronbach's alpha score for the instrument was 0.91, indicating high reliability (Jeffries and Rizzolo 2006).

The Simulation Design Scale (SDS) is a 20‐item scale developed by NLN to assess the implemented simulation designs; five items examine the written “objectives,” materials, and cues needed when simulations are used to guide the students' learning and outcome achievements, four items examine the “support” and provision of support that offered to students in the learning process, five items examine the facilitation and opportunities for students “problem‐solving” abilities, four items examine the constructive “feedback” and reflection provided to students, and last two items examine “fidelity” and realistic environmental factors that provided during learning. Response options for each item using a Likert‐style scale are (1) strongly disagree, (2) disagree, (3) undecided, (4) agree, (5) strongly agree, and (NA) not applicable (Jeffries and Rizzolo 2006). Cronbach's alpha score for the instrument was 0.95, indicating high reliability (Jeffries and Rizzolo 2006).

### 2.4 Ethical Considerations

Upon accessing the online questionnaire, a cover letter informs students about the survey's purpose, objectives, inclusion criteria, time to complete the questionnaire, their role in the survey, and the right to participate or withdraw from the survey at any time. Students were assured that their choice to participate would not affect their education and that their responses would be kept confidential and accessed only by the research team. Finally, a statement informs students that proceeding to complete the questionnaire will be considered as his/her consent to participate in the survey.

### 2.5 Findings

After completing the data collection using Google Forms, data was extracted as an MS Excel sheet and entered into Statistical Package for Social Sciences (SPSS) Version 23.0. It got coded, screened, and scanned for missing data and outliers. No missing data was there as all the questions were mandatory to answer, and outliers were treated by deletion since the number of participants was higher than the needed sample size. Then, statistical analyses were conducted to conclude the results. Participants' demographics were reported using descriptive statistics of frequencies and percentages. Meanwhile, means and standard deviations were used to report the scores of the two main scales (EPQ and SDS) and their subscales. Finally, the rigorous parametric tests, independent samples t‐test, and ANOVA (analysis of variance) with its post hoc testing were used to compare the scores based on the demographic variables. Independent samples t‐test was used to compare the two groups, and ANOVA was used to compare three or more groups (Bowers 2019).

### 2.6 Participants' Demographic Characteristics

Upon completion of data collection, 966 nursing students participated in the study. The average age of students was 21.8 years (SD = 2.16). Female students (*n* = 567, 58.7%) participated more than males; the higher response rate was from students in the second academic year (*n* = 357, 37.0%), and the majority of students were newly enrolled in nursing programmes after completing secondary school (*n* = 756, 78.3%) compared to the Licensed Practical Nurse (LPN) to Registered Nurse (RN) programme. The majority (*n* = 630, 65.2%) of nursing students rated their computer skills as “excellent.” Moreover, a high percentage of them (*n* = 735, 76.1%) agreed that having better computer skills helps in better utilisation of simulation activities. A detailed summary of the participants' demographic characteristics is shown in Table 1.

**TABLE 1:** Participants' Demographic Characteristics (*N* = 966).

Characteristic	Count (%)
Age	
≤ 20 years	442 (45.7%)
> 20 years	525 (54.3%)
Gender	
Female	567 (58.7%)
Male	399 (41.3%)
Academic year	
First	126 (13.0%)
Second	357 (37.0%)
Third	315 (32.6%)
Fourth	168 (17.4%)
Enrolment type	
LPN to RN Program	210 (21.7%)
Admission from secondary school	756 (78.3%)
Computer skills level	
Excellent skills	630 (65.2%)
Average skills	189 (19.6%)
Weak skills	147 (15.2%)
Computer skills affect simulation	
Yes	735 (76.1%)
No	231 (23.9%)

### 2.7 Educational Practices

The mean values of student's responses to the educational practices' questionnaire items are presented in Table 2. The scores are generally homogeneous, with a close range between an average of 3.18 for the lowest item and 3.98 for the highest (out of 5.0 as the highest possible score for the scale and its subscales). The subscale of attitudes and opportunities to have an “Active Learning” experience using simulation had the highest scores (*M* = 3.47 ± 1.17). Nursing students have “High Expectations” (*M* = 4.44 ± 1.05) and perceived good levels of “Collaboration” (*M* = 3.38 ± 0.97). The lowest scores were for the “Diverse Way of Learning” (*M* = 3.18 ± 1.05).

**TABLE 2:** Students' Responses to Educational Practices Questionnaire (Student Version).

Item	Mean	SD
*Active learning*	3.47	0.83
1. I had the opportunity during the simulation activity to discuss the ideas and concepts taught in the course with the teacher and other students.	3.70	1.17
2. I actively participated in the debriefing session after the simulation.	3.30	1.26
3. I had the opportunity to put more thought into my comments during the debriefing session.	3.31	1.21
4. There were enough opportunities in the simulation to find out if I clearly understand the material.	3.38	1.23
5. I learned from the comments made by the teacher before, during, or after the simulation.	3.72	1.11
6. I received cues during the simulation in a timely manner.	3.48	1.13
7. I had the chance to discuss the simulation objectives with my teacher.	3.49	1.28
8. I had the opportunity to discuss ideas and concepts taught in the simulation with my instructor.	3.57	1.05
9. The instructor was able to respond to the individual needs of learners during the simulation.	3.64	1.26
10. Using simulation activities made my learning time more productive.	3.14	1.29
*Collaboration*	3.38	0.97
11. I had the chance to work with my peers during the simulation.	3.51	1.21
12. During the simulation, my peers and I had to work on the clinical situation together.	3.26	1.28
*Diverse Way of Learning*	3.18	1.05
13. The simulation offered a variety of ways in which to learn the material.	3.19	1.17
14. This simulation offered a variety way of assessing my learning.	3.18	1.10
*High Expectations*	3.44	1.05
15. The objectives for the simulation experience were clear and easy to understand.	2.90	1.34
16. My instructor communicated the goals and expectations to accomplish during the simulation.	3.98	1.13

### 2.8 Simulation Design Dimensions

The mean values of students' responses to the simulation design scale are presented in Table 3. The scores were homogenous as well, ranging between an average of 3.41 for the lowest item and 3.87 for the highest (out of 5.0 as the highest possible score for the scale and its subscales). Receiving “Support” during the simulation‐based learning had the highest score (*M* = 3.67 ± 1.3), then having clear “Objectives and Information” (*M* = 3.66 ± 1.22), “Fidelity” also had a high score (*M* = 3.64 ± 1.18) along with improving the abilities of the students to perform “Problem‐Solving” through simulation‐based learning (*M* = 3.62 ± 1.22). Finally, the lowest score was for having “Feedback” about the simulation‐based experience in a constructive and timely manner (*M* = 3.64 ± 1.18).

**TABLE 3:** Students' Responses to Simulation Design Scale (Student Version).

Item	Mean	SD
*Objectives and information*	3.66	1.22
1. There was enough information provided at the beginning of the simulation to provide direction and encouragement.	3.54	1.33
2. I clearly understood the purpose and objectives of the simulation.	3.70	1.21
3. The simulation provided enough information in a clear matter for me to problem‐solve the situation.	3.57	1.21
4. There was enough information provided to me during the simulation.	3.76	1.22
5. The cues were appropriate and geared to promote my understanding.	3.72	1.14
*Support*	3.67	1.30
6. Support was offered in a timely manner.	3.50	1.315
7. My need for help was recognised.	3.65	1.323
8. I felt supported by the teacher's assistance during the simulation.	3.65	1.339
9. I was supported in the learning process.	3.87	1.209
*Problem Solving*	3.62	1.22
10. Independent problem‐solving was facilitated.	3.41	1.244
11. I was encouraged to explore all possibilities of the simulation.	3.85	1.083
12. The simulation was designed for my specific level of knowledge and skills.	3.67	1.181
13. The simulation allowed me the opportunity to prioritise nursing assessments and care.	3.61	1.277
14. The simulation provided me an opportunity to goal set for my patient.	3.57	1.297
*Feedback/Guided Reflection*	3.57	1.23
15. Feedback provided was constructive.	3.43	1.280
16. Feedback was provided in a timely manner.	3.67	1.217
17. The simulation allowed me to analyse my own behaviour and actions.	3.54	1.229
18. There was an opportunity after the simulation to obtain guidance/feedback from the teacher in order to build knowledge to another level.	3.63	1.187
*Fidelity* (*Realism*)	3.64	1.18
19. The scenario resembled a real‐life situation.	3.67	1.199
20. Real life factors, situations, and variables were built into the simulation scenario.	3.61	1.170

### 2.9 Differences in SDS and EPQ Among Students' Groups

The differences among students' groups (based on demographics categories) in the perceptions of simulation design and the utilised educational practices are presented in Table 4. An independent samples t‐test revealed significant differences between the students based on their age, where older students have statistically higher SDS scores (*M* = 3.44 ± 0.82) and EPQ scores (*M* = 3.53 ± 0.64) compared to younger students (*M* = 3.16 ± 0.99 and *M* = 3.29 ± 0.92, respectively).

**TABLE 4:** Comparison of SDS and EPQ scores per students' demographics (*n* = 966).

Variables Profile	SDS		EPQ	
	Mean ± SD	*p*	Mean ± SD	*p*
Age				
20 >	3.16 ± 0.99	< 0.001^a^	3.29 ± 0.92	< 0.001^a^
20 ≤	3.44 ± 0.82		3.53 ± 0.64	
Gender				
Male	3.36 ± 0.88	0.188^a^	3.37 ± 0.83	0.102^a^
Female	3.28 ± 0.93		3.46 ± 0.77	
Academic Year				
First	2.81 ± 1.03	< 0.001^b^	3.04 ± 1.17	< 0.001^b^
Second	3.33 ± 0.88		3.44 ± 0.69	
Third	3.28 ± 0.96		3.36 ± 0.73	
Fourth	3.7 ± 0.53		3.75 ± 0.58	
Enrolment Type				
New Admission	3.14 ± 0.89	< 0.001^a^	3.23 ± 0.75	< 0.001^a^
Bridging Students	3.91 ± 0.71		4.08 ± 0.52	
Computer skills level				
Excellent skills	3.37 ± 1.00	< 0.001^b^	3.54 ± 0.83	< 0.001^b^
Medium skills	3.19 ± 0.80		3.18 ± 0.54	
Weak skills	3.19 ± 0.54		3.17 ± 0.73	
Computer skills affect simulation				
Yes	3.36 ± 0.89	< 0.001^a^	3.50 ± 0.82	< 0.001^a^
No	3.13 ± 0.94		3.13 ± 0.60	

^a^ *t*‐test.

^b^ ANOVA.

The results continue to present that LPN to RN program students have statistically higher SDS scores (*M* = 3.91 ± 0.71) and EPQ scores (*M* = 4.08 ± 0.52) compared to new admission students' scores (*M* = 3.14 ± 0.89 and *M* = 3.23 ± 0.75, respectively). The same results also emerged about students' academic year level. ANOVA Welch's Test with adjusted F ratio and the Games‐Howell post hoc test were conducted (Dalgaard, 2008). The results revealed significant differences between the students based on their academic year level, in which the higher the student's level, the higher the SDS and EPQ scores that were obtained. First‐year students have statistically lower overall SDS scores (*M* = 2.81 ± 1.03) and EPQ scores (*M* = 3.04 ± 1.17) compared to student scores in other year levels. Also, students in the fourth‐year level have statistically higher overall SDS scores (*M* = 3.7 ± 0.53) and EPQ scores (*M* = 3.75 ± 0.58) compared to student scores in other year levels.

The results also revealed a significant difference between the students based on their computer skills level, in which students with higher computer skills have statistically higher SDS scores (*M* = 3.37 ± 1.00) and EPQ scores (*M* = 3.54 ± 0.83) compared to students who have a lower‐rated computer skill. Similarly, students who believed that their computer skills affect simulation use have statistically higher overall SDS scores (*M* = 3.36 ± 0.89) and EPQ scores (*M* = 3.50 ± 0.82) than students who stated that their computer skills do not affect simulation.

## 3 Discussion

Clinical training is integral to nursing curricula, especially when pandemic outbreaks forced nursing schools to cancel clinical placements temporarily, negatively affecting students' ability to conduct accurate clinical assessments and standard procedures (Nabolsi et al. 2021). This study confirms that nursing students in Jordan interact actively with simulation and how it is used, presenting their perceptions about the different dimensions of simulation and its related educational practices. The results concur with findings in other studies worldwide (Hope et al. 2011). Also, the current study confirms the relationship between simulation design and related educational practices. The result is consistent with a meta‐analysis study that found a strong correlation and effect of simulation‐based nursing on students' educational practices (Shorey and Ng 2021).

Nevertheless, successful simulation‐based education requires attention to different dimensions of the simulation design and related educational practices to improve nursing students' learning outcomes (Franklin et al. 2014; Jeffries and Rogers 2009). Jeffries' National League for Nursing (NLN) scales describe the dimensions of the simulation design and educational practices. It underscores the specific features in simulation design and implementation of best educational practices to evaluate and conclude simulation‐based learning in undergraduate nursing education (Cant and Cooper 2017; Jeffries and Rizzolo 2006; Jeffries and Rogers 2009). The NLN framework has been developed based on theoretical and empirical literature to guide the development and assessment of learning processes and the proposed outcomes to be influenced by the degree to which best practices in education are incorporated in the design and implementation of the simulations (Jeffries 2005).

In evaluating the different dimensions of simulation designs in the current study, the results show that the simulation design scale helped assess students' needs and design features. The three simulation designs of “*support*,” “objectives and information,” and “*fidelity*” were highly rated by students. The need for support during simulation‐based educational activities and having clear objectives and information about the applied simulation scenarios helps maximise the benefits of simulation activity (Cant and Cooper 2017; Jeffries and Rogers 2009). On the other hand, despite literature support for higher “*fidelity*” levels of simulation to better “immerse” students in the learning experience, some controversy still exists (O'Shea et al. 2021). In his review study, Adamson (2015) reported that fidelity could be a key simulation characteristic. However, other studies indicated that the simulation activities do not meet students' expectations regarding its realism, especially in healthcare education (Arthur et al. 2013). Pinar reported that high‐fidelity simulation increases students' self‐confidence and decreases their anxiety regarding learning new things, which would improve their educational experiences and practices (Pinar et al. 2015). The authors concluded that this controversy could be referred to the complex nature of healthcare and the involvement of human subjects “clients” whom healthcare professionals need to work with. It differs from other disciplines where simulation can be utilised, avoiding working with human subjects.

In evaluating the educational practices, the current study demonstrated a high level of “*active learning*” dimension compared to the other dimensions (“*high expectations*,” “collaboration,” and “diverse way of learning”). The same tool has been used in different settings and reported comparable results to this study (Farrés‐Tarafa et al. 2020; Franklin et al. 2014; Unver et al. 2017). Simulation, as a well‐developed learning strategy, has been widely used in nursing classes and is matched with many learning theories—such as adult learning theory‐ which are keen to promote students' active learning practices (Clinard and Dudding 2019). In simulation settings, students feel they are active learners because they can develop their skills in a supportive and risk‐free environment and become more confident in working with critical situations (Cook et al. 2011; Jansen 2015).

In the current study, senior students had higher scores on SDS and EPQ scales, which finding is consistent with a study conducted in Saudi Arabia (Aljohani et al. 2019). However, it was in contrast to another study where the student's age and academic year were inversely correlated with students' SDS and EPQ scores (Albagawi et al. 2021). The adult learning theory can explain the results. Adult learners are more ready and motivated to learn when there is a clear reason and goal to develop their path. Also, they want to be actively involved in the learning process and use their accumulative experiences to draw knowledge (Knowles 1978). The current study confirms these assumptions, as simulation‐based education targets students' interests, involves them in the learning process, and helps them to make new decisions based on their previous knowledge and experiences.

The current study supports the focus of involving the “active learning” educational practice along with a simulation‐based learning experience that includes enough “support” to students' learning experience, a clear definition ensuring students' comprehension of “objectives and information” of the educational activity, combined with the highest possible “fidelity” level. Utilising the best simulation design with a focus on better educational practices may enhance the utilisation of simulation in undergraduate nursing education. Currently, it is reported by Masuda and colleagues (2021) at their national level survey in Japan that simulation is “underused” for such reasons. Simulation‐based learning is “mature” enough and “available” to undergraduate nursing education, but more work is still needed for better utilisation and enhanced learning outcomes (Aebersold 2018).

### 3.1 Study Limitations and Recommendations

Despite the authors' attempts to overcome any weaknesses in the study, a few limitations were faced. The study's descriptive design limits the ability to fully disclose the student's lived experience in using simulation‐based education and evaluate the dimensions of the related educational practices. Future qualitative studies may help better understand nursing students' lived experiences. Moreover, more situational factors may affect the study results, which may need further exploration and control in future studies. Such factors include the instructors' competency in running simulation technology and tools, the level of fidelity used, and the availability of the different modules for the various courses at the undergraduate level. As a result of the study, it is also recommended to put in strategies to involve early years students to utilise simulation better and make them feel more comfortable in using simulation, especially the recommended high‐fidelity designs.

## 4 Conclusions

Simulation‐based education is an essential component of nursing education. It needs to be incorporated at the different levels of nursing programs while focusing on increasing the support to students in simulation laboratories, helping students to comprehend and work toward achieving the objectives of the simulation activities and expanding the availability of high‐fidelity simulations. Active learning strategies should also be implemented to enhance students' opportunities to participate in simulation‐based educational activities.

## Author Contributions

M.A., M.R.A., E.H.O., H.O., A.J.N. made substantial contributions to conception and design, or acquisition of data, or analysis and interpretation of data. M.A., M.R.A., E.H.O., H.O., A.J.N. involved in drafting the manuscript or revising it critically for important intellectual content. M.A., M.R.A., E.H.O., H.O., A.J.N. given final approval of the version to be published. Each author should have participated sufficiently in the work to take public responsibility for appropriate portions of the content. M.A. agreed to be accountable for all aspects of the work in ensuring that questions related to the accuracy or integrity of any part of the work are appropriately investigated and resolved.

## Ethics Statement

Ethical approval for this study was granted by The University of Jordan, Faculty of Nursing IRB in October 2022.

## Consent

Participants received a detailed information sheet with the survey link, ensuring informed consent. Data confidentiality and participant anonymity are strictly maintained, adhering to the Declaration of Helsinki and relevant guidelines.

## Conflicts of Interest

The authors declare no conflicts of interest.

## Supporting information


Data S1.

## Data Availability

The authors confirm that the data supporting the findings of this study are available.
