# Supplementary material for: Optimising Educational Practices in Simulation‐Based Nursing Education: Results of A National Survey
Source: Nurs Open. 2025 Apr 23;12(4):e70172. doi: 10.1002/nop2.70172 (PMC12018161; doi:10.1002/nop2.70172)
Supplement: Supplementary file 1 — Data S1. [file NOP2-12-e70172-s001.docx]

STROBE Statement—Checklist of items that should be included in reports of ***cross-sectional studies***

| **Item** | Item No | Recommendation | Status |
| --- | --- | --- | --- |
| **Title and abstract** | 1 | (*a*) Indicate the study’s design with a commonly used term in the title or the abstract | Met at the title and abstract Page 1 |
|  |  | (*b*) Provide in the abstract an informative and balanced summary of what was done and what was found | Met  Page 1 |
| Introduction | | | |
| Background/rationale | 2 | Explain the scientific background and rationale for the investigation being reported | Completed  Page 2 and 3 |
| Objectives | 3 | State-specific objectives, including any prespecified hypotheses | As stated at the abstract and the end of the introduction section  Page 4 |
| Methods | | | |
| Study design | 4 | Present key elements of study design early in the paper | Met  Page 4 |
| Setting | 5 | Describe the setting, locations, and relevant dates, including periods of recruitment, exposure, follow-up, and data collection | Met  Page 4 |
| Participants | 6 | (*a*) Give the eligibility criteria, and the sources and methods of selection of participants | Met  Page 4 |
| Variables | 7 | Clearly define all outcomes, exposures, predictors, potential confounders, and effect modifiers. Give diagnostic criteria, if applicable | Met  Page 5 (Instruments) |
| Data sources/ measurement | 8* | For each variable of interest, give sources of data and details of methods of assessment (measurement). Describe comparability of assessment methods if there is more than one group | Met  Page 5 (Instruments) |
| Bias | 9 | Describe any efforts to address potential sources of bias | Met  Page 6 |
| Study size | 10 | Explain how the study size was arrived at | Met  Page 5 and 6 |
| Quantitative variables | 11 | Explain how quantitative variables were handled in the analyses. If applicable, describe which groupings were chosen and why | Met  Page 5 (Instruments) |
| Statistical methods | 12 | (*a*) Describe all statistical methods, including those used to control for confounding | Met  Page 6 |
|  |  | (*b*) Describe any methods used to examine subgroups and interactions | Met  Page 6 |
|  |  | (*c*) Explain how missing data were addressed |  |
|  |  | (*d*) If applicable, describe analytical methods taking account of sampling strategy | Met  Page 6 |
|  |  | (*e*) Describe any sensitivity analyses | Not applicable |
| Results | | | |
| Participants | 13* | (a) Report numbers of individuals at each stage of study—eg numbers potentially eligible, examined for eligibility, confirmed eligible, included in the study, completing follow-up, and analysed | Nor Applicable |
|  |  | (b) Give reasons for non-participation at each stage | Not applicable |
|  |  | (c) Consider use of a flow diagram | Considered  Not Applicable |
| Descriptive data | 14* | (a) Give characteristics of study participants (eg demographic, clinical, social) and information on exposures and potential confounders | Met  Page 7 |
|  |  | (b) Indicate number of participants with missing data for each variable of interest | Met  Page 6 |
| Outcome data | 15* | Report numbers of outcome events or summary measures | Met  Pages 7 and 8 |
| Main results | 16 | (*a*) Give unadjusted estimates and, if applicable, confounder-adjusted estimates and their precision (eg, 95% confidence interval). Make clear which confounders were adjusted for and why they were included | No adjustments were needed.  No applicable |
|  |  | (*b*) Report category boundaries when continuous variables were categorized | Met  Table 1, Page7 |
|  |  | (*c*) If relevant, consider translating estimates of relative risk into absolute risk for a meaningful time period | Not applicable |
| Other analyses | 17 | Report other analyses done—eg analyses of subgroups and interactions, and sensitivity analyses | Not applicable |
| Discussion | | | |
| Key results | 18 | Summarise key results with reference to study objectives | Met  Pages 6-9 |
| Limitations | 19 | Discuss limitations of the study, taking into account sources of potential bias or imprecision. Discuss both direction and magnitude of any potential bias | Met  Page 12 |
| Interpretation | 20 | Give a cautious overall interpretation of results considering objectives, limitations, multiplicity of analyses, results from similar studies, and other relevant evidence | Met  Page 12 (Conclusion) |
| Generalisability | 21 | Discuss the generalisability (external validity) of the study results | Met  Page12 |
| Other information | | | |
| Funding | 22 | Give the source of funding and the role of the funders for the present study and, if applicable, for the original study on which the present article is based | Met  Page 13 (Declarations) |

*Give information separately for exposed and unexposed groups.

**Note:** An Explanation and Elaboration article discusses each checklist item and gives methodological background and published examples of transparent reporting. The STROBE checklist is best used in conjunction with this article (freely available on the Web sites of PLoS Medicine at http://www.plosmedicine.org/, Annals of Internal Medicine at http://www.annals.org/, and Epidemiology at http://www.epidem.com/). Information on the STROBE Initiative is available at www.strobe-statement.org.
